# The relationship between species richness and aboveground biomass in a primary *Pinus kesiya* forest of Yunnan, southwestern China

**DOI:** 10.1371/journal.pone.0191140

**Published:** 2018-01-11

**Authors:** Shuaifeng Li, Xuedong Lang, Wande Liu, Guanglong Ou, Hui Xu, Jianrong Su

**Affiliations:** 1 Research Institute of Resource Insects, Chinese Academy of Forestry, Kunming, China; 2 Pu`er Forest Eco-system Research Station, China’s State Forestry Administration, Kunming, China; 3 Key laboratory of State Forest Administration on Biodiversity Conservation in Southwest China, Southwest Forestry University, Kunming, China; Tennessee State University, UNITED STATES

## Abstract

The relationship between biodiversity and biomass is an essential element of the natural ecosystem functioning. Our research aims at assessing the effects of species richness on the aboveground biomass and the ecological driver of this relationship in a primary *Pinus kesiya* forest. We sampled 112 plots of the primary *P*. *kesiya* forests in Yunnan Province. The general linear model and the structural equation model were used to estimate relative effects of multivariate factors among aboveground biomass, species richness and the other explanatory variables, including climate moisture index, soil nutrient regime and stand age. We found a positive linear regression relationship between the species richness and aboveground biomass using ordinary least squares regressions. The species richness and soil nutrient regime had no direct significant effect on aboveground biomass. However, the climate moisture index and stand age had direct effects on aboveground biomass. The climate moisture index could be a better link to mediate the relationship between species richness and aboveground biomass. The species richness affected aboveground biomass which was mediated by the climate moisture index. Stand age had direct and indirect effects on aboveground biomass through the climate moisture index. Our results revealed that climate moisture index had a positive feedback in the relationship between species richness and aboveground biomass, which played an important role in a link between biodiversity maintenance and ecosystem functioning. Meanwhile, climate moisture index not only affected positively on aboveground biomass, but also indirectly through species richness. The information would be helpful in understanding the biodiversity-aboveground biomass relationship of a primary *P*. *kesiya* forest and for forest management.

## Introduction

Biodiversity and biomass are two critical variables in the plant community ecosystem [1]. Biodiversity declines have led to widespread concern about the loss of ecosystem function resulting from human disturbance including deforestation and afforestation under the background of global climate change [2]. The biodiversity-biomass relationship has become a major ecological focus worldwide over recent decades [3, 4]. However, the relationship between species diversity and biomass (sometimes instead of productivity) has led to more controversial conclusions: (1) biomass increased with species diversity, (2) biomass decreased with species diversity, and (3) no definite change [5, 6]. The original studies discussed relationships in experimental communities, especially in fast-growing ecosystems with simple community structure, such as grasslands, meadows, and wetlands [7]. The ecologists have discovered that increasing plant diversity tends to be correlated with higher community productivity since the 1990s [8, 9].

Recently, equivocal findings have been obtained from existing studies with respect to the fundamental relationship between plant species diversity and biomass or productivity. Most studies have found that biodiversity could increase community biomass or productivity, whether in simple grassland ecosystems or in complex natural forest ecosystems [4, 10, 11]. A few studies found that lower biodiversity levels are associated with higher biomass production [12, 13]. Others have found few consistent relationships in natural ecosystems [9, 14, 15]. The unimodal curve was the common variation tendency found between biodiversity and biomass in the different natural ecosystems using observation methods [16], but no findings depicted consistent causal mechanisms.

The driving mechanisms of the biodiversity-biomass variations may be explained by the sampling effect and the complementary effect, both highly contingent on our understanding of complex natural communities and spatial variation scales [2]. Generally speaking, the sampling effect could illustrate that the most productive species will ultimately dominate the proportion of community biomass, while the complementary effect could enhance a functioning process such as productivity through niche partitioning and interspecific facilitation, leading to more utilization of resources [10, 17]. The sampling effect and the complementary effect are not mutually exclusive, and both mechanisms will likely affect biomass and productivity. The intensity of responses had larger variation in differing environments and the complementary effect accounted for a large proportion of explanatory ability in large-scale patterns [15, 18].

In the case of forest ecosystems, the hypothesis that increasing tree species diversity translates into elevated biomass accumulation is difficult to evaluate through experimental manipulations such as those conducted in grassland ecosystems. Because of the much slower growth of trees, it is difficult to explore the ecological impact on the biodiversity-biomass relationship. Rather, it is more feasible to explore relationships through meta-analysis of existing datasets. Multivariate analysis techniques have been used to develop understanding of biodiversity-biomass relationships [17, 19]. The relationship between plant species diversity and biomass accumulation has been examined in different types of forests using a range of statistical methods. For example, Zhang and Chen found a positive correlation between diversity and aboveground biomass in a natural temperate spruce and pine forest [17]. In contrast, Jerzy and Anna found a weak negative relationship between species diversity and biomass accumulation in a pine forest of Europe [13]. One possible explanation for these differences among existing studies is that the most competitive tree species may not always be the most productive and complementary effect on both environmental conditions and species functional characteristics [20]. Interactions between species and the environment and between different species can shape the nature of the species diversity-biomass relationship [21]. Climate factors limited the productivity of the community in a larger scale, while hygrothermal index could explain a larger proportion of pine forest productivity [22]. Simultaneously, plant species diversity usually increases monotonically with the climate variables increase, and climate factors become important driving mediation between biodiversity and forest biomass [23]. Furthermore, the soil nutrient regime has been demonstrated to alter the strength of the biodiversity-biomass relationship [18, 24].

*Pinus kesiya* Royle ex Gord is an important subtropical mixed pine forest ecosystem in the southern region of Yunnan Province because of its fast growth and high timber production. The mixed pine forest encompasses an area of 49.04×10^4^ ha and offers an important source of resin and timber for local communities due to its rapid growth, excellent material quality and high resin production. The natural mixed pine forests in this region have high species richness associated with immigration from nearby monsoon forests [25]. The vast majority of mixed pine forests throughout this larger region have been subject to commercial logging and conversion to plantations or agriculture resulting in species loss and reductions in stored carbon [26].

Forest productivity had a strong correlation with biomass after considering the effects of stand age [23], but most of earlier forest studies have confirmed that productivity could be replaced of biomass in the biodiversity-biomass relationship [3]. We substituted forest aboveground biomass for productivity. To evaluate the diversity-biomass relationship hypothesis in stand level of this forest type, we examined the relationships between aboveground biomass, species richness, stand age, the soil nutrient regime, and the climate moisture index in a *P*. *kesiya* primary forest using general linear models (GLMs) and structural equation models (SEMs) [11,27]. The SEM approach facilitates the quantitative analysis of specific relationships outlined in causal diagrams which has utility in elucidating interacting networks of controlling factors which provided important insights into the links between biodiversity and aboveground biomass [24, 27, 28]. We specified the following compound pathways of multivariate models referring to Zhang and Chen [11]: (1) Nutrient regime and stand age affects aboveground biomass, species richness and climate moisture index respectively, (2) climate moisture index acts on the positive relationship between aboveground biomass and tree species diversity. The primary objective of the research was to determine whether aboveground biomass is positively correlated with tree species diversity and whether any of the fore-mentioned causal mechanisms gives rise to variation between species richness and aboveground biomass.

## Materials and methods

### Study site and data set

**Fig 1:**
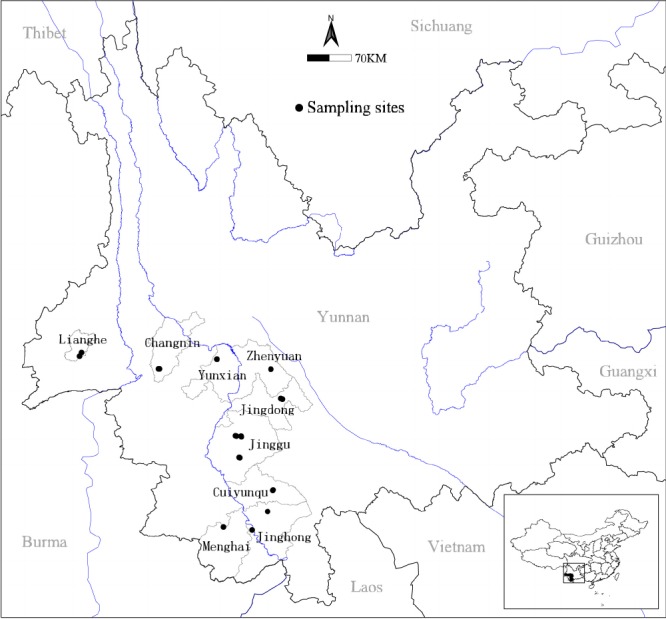
The distribution of 112 plots inventoried in the *Pinus kesiya* primary forest by using ArcGIS 9.3(ESRI,Redlands,CA,USA;http://www.esri.com).

The field plots are distributed in 9 counties including Simao, Jinghong, Menghai, Jinggu, Zhenyuan, Jingdong, Yunxian, Changning and Lianghe of Sounthwestern Yunnan Province, China, which range from 22°11´ to 24°38´ N latitude and from 22°11´ to 24°38´ E longitude, and altitude range from 900 m to 1800 m (Fig 1). The climate is characterized by distinct wet and dry seasons as southern subtropical mountain monsoon. The mean annual temperature and precipitation range from 14.9°C to 21.8°C and 904.7 mm to 1626.5 mm respectively [25]. The plots represented mainly the mixed pine forest (i.e. the forest is dominated by *Pinus keisya* along with evergreen broadleaf trees such as species of *Schima wallichii*, *Castanopsis echidnocarpa* and *Lithocarpus fenestratus*) with different altitudes, soil nutrient regime, topoclimate and community types which are randomly established in these region.

We selected 112 field plots from January to March of each year during 2012–2014 and each plot size is 20 m×20 m (400 m^2^) referring to the demand of *Yunnan vegetation* [29]. The plots were separated by at least 500 m and arranged on a grid across almost all the mixed pine forest—region of Yunnan. All woody species including tree, liana and shrub, were identified and their diameter at breast height (DBH ≥ 1cm) and height were measured in each plot. Simultaneously, a list of shrub and herbaceous species has been sampling. The community structure is simple with the single dominant stand in the primary *P*. *kesiya* forests. The field plots showed more or less anthropogenic disturbance including of logging for timber, rosin and wild mushroom collecting, and most plots located in the programs of Natural Forest Protection Reserve. *P*. *kesiya* primary forest originated from natural regeneration of monsoon evergreen broadleaf forest and cutover land of coniferous forest. Most of *P*. *kesiya* primary forests were even-aged stand. *P*. *kesiya* is as the absolutely dominant species in the forest overstory, and accompanied by some other tree species including *Schima wallichii*, *Castanopsis hystrix*, *Castanopsis echidnocarpa*, *Lithocarpus fenestratus*, *Vaccinium exaristatum*, *Wendlandia tinctoria* subsp. *intermedia*, *Glochidion lanceolarium*, *Aporusa villosa*, *Machilus rufipes*, *Anneslea fragrans*. We also recorded shrubs species including *Glochidion eriocarpum*, *Melastoma affine*, *Canthium horridum*, *Ficus hirta* and herbs such as *Hedychium coccineum*, *Scleria herbecarpa*, *Zingiber striolatum*, *Eupatorium adenophorum* and *Dicranopteris pedata* in the forest understory.

### Fieldwork permission

The project had been officially registered at the Research Institute of Resource Insects, Chinese Academy of Forestry. Pu’er forestry Bureau issued the permission to conduct our study for all locations. The fieldwork did not involve endangered or protected species.

### Aboveground biomass estimate

We applied a non-destructive method to estimate the aboveground biomass in our study. 115 tree species were recorded in all field plots and classified as 10 types for more accurate aboveground biomass estimation: (1) *P*. *kesiya* [30], (2) *Schima wallichii* and aboveground biomass model was built by ourselves, (3) *Castanopsis hystrix* [31], (4) *Castanopsis echidnocarpa* [32], (5) *Betula alnoides* [33], (6) *Rhus chinensis* [34], (7) *Alnus cremastogyne* [35], (8) other mixed tree species [36], (9) shrubs and small trees [37] and (10) lianas [38]. DBH and the height of each individual tree were used to estimate the aboveground biomass using different biomass allometric equations (Table 1). Total aboveground biomass production in the plots was obtained by summing the biomass of all the standing trees and aboveground biomass of each plot can be transformed for per ha (t·ha^-1^).

**Table 1:** Biomass allometric equations of each component of *Pinus kesiya* and other broadleaf species.

Number	Tree species/group	Component	Allometric equation*
1	*Pinus kesiya*	trunk	*Y =* 0.0808*D*^2.5374^
		branch	*Y =* 0.0007*D*^3.4663^
		needle	*Y =* 0.0015*D*^2.504^
2	*Schima wallichii*	Aboveground	*Y =* 0.24*D*^2.072^
3	*Castanopsis hystrix*	trunk	*Y =* 0.06411*(D*^2^*H)*^0.8699^
		Bark	*Y =* 0.0105*(D*^*2*^*H)*^0.8246^
		branch	*Y =* 0.00011*(D*^*2*^*H)*^1.3949^
		Leaf	*Y = 0*.*0000028(D*^*2*^*H)*^1.6052^
4	*Castanopsis echidnocarpa*	trunk	*y* = 1.33258×10^−2^ (1.8224+*D)*^3^
		branch	*y* = 0.6053+1.0218×10^-3^*D*^3^
		Leaf	*y* = 0.5028+2.9591×10^-4^*D*^4^
5	*Betula alnoides*	trunk	*Y = 0*.*15D*^2.1969^
		branch	*Y = 0*.*0313D*^2.2118^
		Leaf	*Y = 0*.*0094D*^2.0184^
6	*Rhus chinensis*	Above ground	*Y = 0*.*3D*^2.077^
7	*Alnus cremastogyne*	trunk	*Y* = 0.027388(D^2^H)^0.898869^
		bark	*Y* = 0.012101(D^2^H)^0.854295^
		branch	*Y* = 0.014972(D^2^H)^0.875639^
		leaf	*Y* = 0.010593(D^2^H)^0.813953^
8	Other trees	Aboveground	*Y = 0*.*1381D*^2.3771^
9	Shrub and small tree	Aboveground	LN(*Y*) *=* 3.5+1.65LN(*D*)*+*0.842LN(*H)*
10	Liana	Aboveground	*y =* 0.074(*D*^2^*L*)0.8495

*Y is the biomass of the tree component (kg), *D* is the diameter at breast height (cm) and *H* is the tree height (m) and *L* is the length of liana (m).

### Species richness

Species richness is generally used to measure the biodiversity in plant community [2]. In our study, species richness was measured as the number of species including tree, liana and shrub species in a plot unit.

### Climate

The annual mean precipitation and monthly temperature in each plot were obtained according to Climate AP software [39]. The climate AP software requires the latitude, longitude and altitude in each field plot, and then generates the climate factors which had a good fit on the basis of 137 meteorological stations in Yunnan Province. We selected the climate moisture index (CMI, mm, annual precipitation minus annual potential evapotranspiration), since higher climate moisture index values could better represent higher water availability for plants [23].

### Stand age

Stand age (years) for each plot was determined by mean value of the oldest three *P*. *kesiya* inside or outside the plot at each plot site, which used as a conservative estimate of stand age [11], which were calculated by the allometric growth model of tree age-DBH of *P*. *kesiya* according to 91 sample trees with the ranges of the tree age from 8 to 82. The equation of tree age (y) use the following formula: *y* = 3.326*DBH*^0.733^ (*R*^2^ = 0.802, *P*<0.001, *F* = 357.323, n = 90).

### Soil nutrient regime

We collected the soil samples with deep 0–20 cm of soil surface and analyzed the soil pH, soil organic matter, total nitrogen, total phosphorus, total potassium, available nitrogen, available phosphorus and available potassium, which can represent the soil nutrient regime to maximum extent. We selected available phosphorus as a trait of soil nutrient regime which is a better indicator for the plant growth in the red soil types of subtropical and tropical zone.

### Statistical analysis

All variables were tested for normality using a Shapiro-Wilk goodness-of-fit test. These variables violated the normality assumption which needed log transformation to ensure that predicted values of all quantities would be positive including species richness, aboveground biomass and climate moisture index except stand age and soil nutrient regime for our field data analysis. Simultaneously, to aid in construction of SEM, we examined the bivariate relationships between each hypothesized causal path according to our framework hypotheses [28]. Firstly, Simple linear regression and polynomial regressions by adding a quadratic polynomial term fit better in each pair of variables, which can assess whether aboveground biomass is dependent on tree species richness [11]. Secondly, to account for other environmental differences, we used general linear models (GLMs) to explain aboveground biomass, climate moisture index and species richness using environmental factors as well as species richness respectively.

We specified a meta-model based on the known theoretical framework including the hypothesized multiple paths predicted by the multivariate biodiversity-biomass hypothesis [11, 28]. The nonparametric Bollen–Stine bootstrapping estimations were used for improved robustness of our SEM for addressing the potential issues from nonlinear and remaining univariate non-normality after transformations. Recommended chi-square tests, root mean square error of approximation (RMSEA) and goodness-of-fit index (GFI) have used to evaluate the model fit of all SEMs [11, 40]. A chi-square with a *P* > 0.05 indicates that the observed and expected covariance matrices are not statistically different; the values of RMSEA and GFI ranging < 0.05 and > 0.95 respectively, suggest a good model fit [11,40]. The significant path coefficient for directional paths (single-headed arrows) indicates statistically significant in the causal relationship. Furthermore, the path coefficient, standardized for comparison between pathways, can be a measure for the sensitivity of dependent variable to the predictor [40]. To further enhance the interpretation of SEM results, the total effects of a given exogenous variable on aboveground biomass was estimated by adding the direct standardized effect and the indirect standardized effect [11].The SEMs were implemented using the “lavaan” package [40]and all statistic analyses were performed with R 3.3.1 (R Development Core Team 2016).

## Results

### Relationships between species richness and aboveground biomass

**Fig 2:**
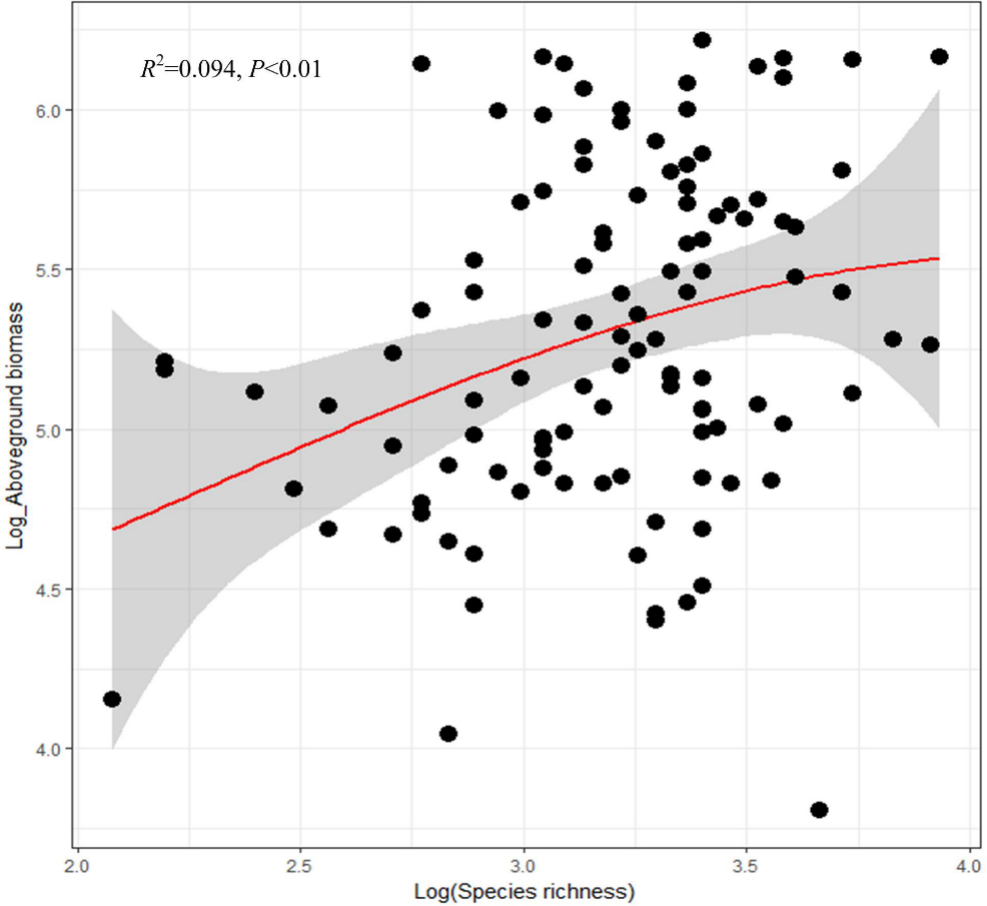
Relationship between species richness and aboveground biomass in a primary *Pinus kesiya* forest. The red solid line is from multiple OLS regression by adding the cubic term. Gray shaded areas show 95% confidence interval of the fit.

We analyzed 112 plots and a total of 249 woody species were recorded. The species richness is 26 ranging from 8 to 51 (S1 Table, Shapiro-Wilk test: *W* = 0.951, *P*<0.001). The aboveground biomass ranged from 54.26 to 489.13 t·ha^-1^ (Shapiro-Wilk test: *W* = 0.906, *P*<0.001). There was a significant positive linear relationship between aboveground biomass and species richness as well as species richness, which conducted ordinary least squares (OLS) regressions by adding the cubic term (Fig 2). The aboveground biomass increased with the species richness increase.

### The effects of species richness and abiotic factors on aboveground biomass

The relationship between species richness, climate moisture index, soil nutrient regime, stand age and aboveground biomass showed different response. We applied the GLMs to examine the combined effect among aboveground biomass, species richness, climate moisture index, soil nutrient regime and stand age (Table 2). The stand age was the most important driver in the aboveground biomass and 63.3% of the variations were accounted for the relationship between aboveground biomass and species richness as well as abiotic factors. Simultaneously, climate moisture index had a positive effect on the aboveground biomass and soil nutrient regime had a negative effect on the aboveground biomass. Species richness had no influence on the model predictions. Climate moisture index represented for 34.81% variation in a GLM model predictions which had a significant influence on the stand age and species richness. Species richness could increase with better stand age and climate moisture index. The stand age and climate moisture index were better links between species richness and aboveground biomass as mediation.

**Table 2:** Summary of the general linear models (GLMs) for the relationships between the endogenous variables and predictor variables, each variable separately analyzed.

Endogenous variables	Sources	Estimate	SE	t-value	Significance Pr(>|t|)	MS	F-value	Significance Pr(>F)	VIF
AGB	Species richness	0.027	0.102	0.265	0.792	2.859	26.793	<0.001	1.315
	CMI	1.275	0.477	2.674	<0.01	5.551	52.027	<0.001	1.534
	Nutrient regime	-0.336	0.165	-2.043	<0.05	4.773	44.733	<0.001	1.438
	Stand age	1.603	0.205	7.812	<0.001	5.511	61.02	<0.001	1.671
Multiple R^2^ = 0.633; residual SE 0.3266 on 107 d.f.									
CMI	Species richness	0.089	0.019	4.727	<0.001	0.168	38.664	<0.001	1.09
	Nutrient regime	-0.024	0.033	-0.734	0.465	0.037	8.478	<0.01	1.43
	Stand age	0.128	0.04	3.245	<0.01	0.046	10.531	<0.01	1.522
Multiple *R*^2^ = 0.3481; residual SE 0.065 on 107 d.f.									
Species richness	Nutrient regime	0.009	0.169	0.054	0.957	0.317	2.791	0.098	1.43
	Stand age	0.518	0.196	2.645	<0.01	0.794	0.009	<0.01	1.43
Multiple *R*^2^ = 0.082; residual SE 0.337 on 107 d.f.									

*df*, degree of freedom; MS, mean square; SE, standard errors; VIF, variance inflation factor.

### Structural equation modeling

**Fig 3:**
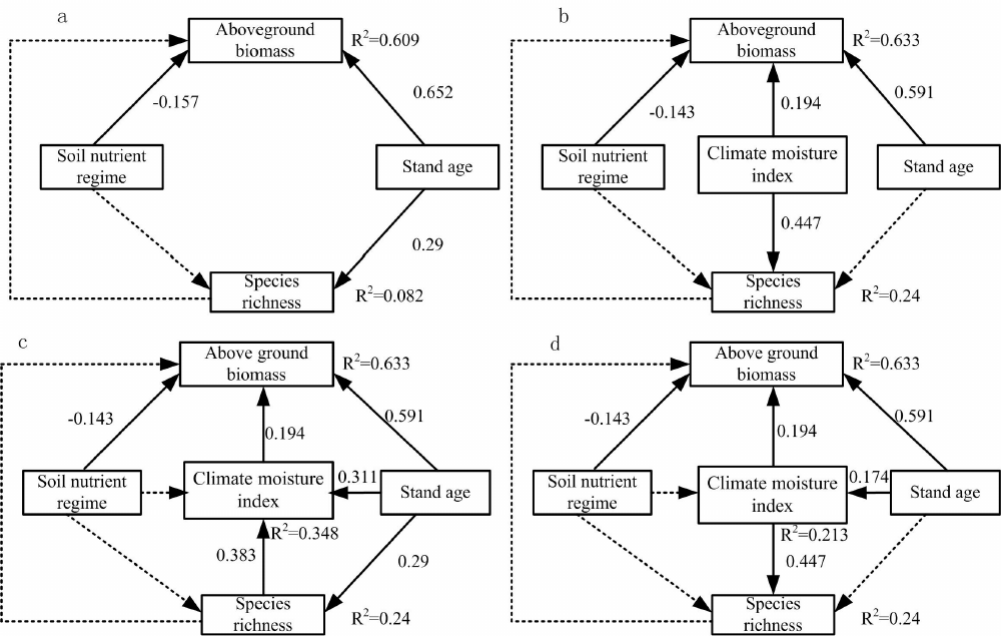
Structural equation models linking aboveground biomass and species richness in the primary *Pinus kesiya* forest. (a) Effects of species richness, soil nutrient regime and stand age on aboveground biomass. (b) Effects of species richness, soil nutrient regime, stand age and climate moisture index on aboveground biomass. (c) and (d) The model with climate moisture index as the linking mechanism. The coefficients are standardized prediction coefficients indicate each path. Solid lines represent significant paths (*P*<0.05) and dash lines indicate non-significant paths (*P*≥0.05).

The four models were used to analyze the relationship between species richness and aboveground biomass and infer the direct and indirect effects of stand age, climate moisture index and soil nutrient regime. The model without climate moisture index as a predictor had a good fit to the data (*x*^2^ = 2.535, *d*.*f*. = 2, *P* = 0.282; *RMSEA* = 0.049; *GFI* = 0.999, Fig 3A). The species richness had no direct effect on aboveground biomass. Meantime, aboveground biomass increased with stand age and climate moisture index showing a positive direct effect on aboveground biomass (Table 3). The soil nutrient regime had a direct effect on aboveground biomass with a negative influence. The model including of climate moisture index still had a better prediction for the links between species richness and aboveground biomass (*x*^2^ = 0.607, *d*.*f*. = 2, *P* = 0.738; *RMSEA*<0.001; *GFI* = 1, Fig 3B). The better hydrothermal condition could increase the aboveground biomass size. The links between species richness and aboveground biomass could be mediated with climate moisture index.

**Table 3:** Direct, indirect and total standardized effects on AGB based on structural equation models.

SEM model	Predictor	Pathway to above-ground biomass	effect
A model in Fig 2A	Species richness	Direct	0.092
	Nutrient regime	Direct	-0.157
		Indirect through species diversity	0.001
		Total effect	-0.156
	Stand age	Direct	0.652**
		Indirect through species richness	0.027
		Total effect	0.678***
A model in Fig 2B	Species richness	Direct	0.018
	Nutrient regime	Direct	-0.143*
		Indirect through species richness	0.001
		Total effect	-0.143*
	Stand age	Direct	0.591***
		Indirect through species richness	0.002
		Total effect	0.593***
	CMI	Direct	0.194**
		Indirect through species richness	0.008
		Total effect	0.202**
B model in Fig 2C	CMI	Direct	0.194**
	Species richness	Direct	0.018
		Indirect through CMI	0.074*
		Total effect	0.92
	Nutrient regime	Direct	-0.143
		Indirect through CMI	-0.013
		Indirect through species richness	-0.001
		Total effect	-0.157
	Stand age	Direct	0.591***
		Indirect through CMI	0.06*
		Indirect through species richness	0.005
		Total effect	0.657***
C model in Fig 2D	CMI	Direct	0.194**
		Indirect through species richness	0.008
		Total effect	0.202***
	Species richness	Direct	0.018
	Nutrient regime	Direct	-0.143*
		Indirect through CMI	-0.013
		Indirect through species richness	0.001
		Total effect	-0.156*
	Stand age	Direct	0.591***
		Indirect through CMI	0.082*
		Indirect through species richness	0.002
		Total effect	0.675***

Significant effects are at *P*<0.05(*), *P*<0.01(**), and *P*<0.001(***).

Next, we added more path analysis including climate moisture index in the full model as a predictor had a good fit to the data (*x*^2^ = 7.287, *d*.*f*. = 3, *P* = 0.063; *RMSEA* = 0.043; *GFI* = 0.999, Fig 3C). Climate moisture index had a positive direct effect on aboveground biomass. The direct path between aboveground biomass and species richness as well as soil nutrient regime became insignificant, but positive effects appeared on between aboveground biomass and stand age as well as climate moisture index (Table 3). Additional total effects of the species richness were realized via changes of climate moisture index. We altered the direction of the path between climate moisture and species richness, whereby the fitting degree of the SEM model became better than that of the third model (*x*^2^ = 5.428, *d*.*f*. = 3, *P* = 0.155; *RMSEA* = 0.096; *GFI* = 0.999, Fig 3D). Like the model in Fig 3C, stand age and climate moisture index had significant indirect effect on aboveground biomass. Climate moisture index had an indirect effect on the aboveground biomass through the stand age. Species richness had no significant effect on aboveground biomass, and soil nutrient regime had a negative significant effect on aboveground biomass.

## Discussion

Our results effectively exhibited a complex and highly variable relationship between species richness and aboveground biomass by employing 112 plots within a primary *P*. *kesiya* forest. If we only considered species richness and the aboveground biomass, we found that the positive linear regression appeared in the species richness-aboveground biomass relationship. The species richness was important for driving power lending to clear differences in aboveground biomass change, and these results are confirmative of a multitude of previous studies showing that biodiversity had an effect on biomass production [11, 16]. In contrast, other pine forest studies generally support the finding from experimental grasslands in a large scale [41].

We found remarkable reasons in the potential mechanisms driving the effect responses, which might be the result of the species diversity per se, or the addition of different functional groups with increased resource partitioning [2,16], such as some productive tree species depending on sampling effect. In our study, as a pioneer species in the tropical region of Yunnan Province, *P*. *kesiya* has the ability to become a dominant tree species in the mixed pine forest and accumulate biomass in a short time [25,26]. Alternatively, the amount of *P*. *kesiya* might be a main source of aboveground biomass increase with species richness. Addition of productive tree species may play an important role in the diversity-biomass relationship which can be explained by the “sampling effects” found to some extent in the subtropical fixed pine forest [18]. Simultaneously, understory species composition showed clear interregional scale differences. Sometimes, the understory conditions have the characteristic of more light and a dry environment, as some sun plants were able to occupy, contributing to more woody production.

Nevertheless, we obtained the interesting finding that species richness had an indirect affect on aboveground biomass as a potential maintenance mechanism in a primary *P*. *kesiya* forest when considered the different abotic factors. The stand age and climate moisture index were important influence factors on the aboveground biomass, but climate moisture index was a better mediation in the links between species richness and aboveground biomass. Aboveground biomass may be influenced indirectly by climate moisture index and soil nutrient regime through species richness according to the multivariate analysis. We can explain it preferably by using a complementary effect [7]. The reason is that signs of logging or resin tapping and other disturbances appear in the subtropical primary *P*. *kesiya* forests, and will lead to larger pine disappear gradually and generate forest gaps which make for survival and settlement of other tree species. Simultaneously, some evergreen broadleaf species have important functional trait (such as sprouting) for a better tree regeneration strategy in this forest community [25]. The shade-tolerant tree species became common dominant components with a better resource utilization rate by the niche differentiation and adaptation to environmental conditions. Canopy tree species diversity increased strongly with regional productivity [22]. A positive biodiversity-biomass relationship appeared in the tropical fixed pine forest. *P*. *kesiya* is likely to give rise to greater numbers of species in the intraspecific assemblages as a particular species, where predation and competition become weaker. Previous studies showed that pine tree abundance had a negative impact on understory biomass production through light, water and soil nutrients, so pine trees had a strong inhibitory effect on the abundance of understory plants, which in turn led to lower understory species richness [28]. Because of this, strong higher upper storey and sub-storey enhanced community vertical structure in the mixed pine forest and *P*. *kesiya* dispersed mainly in the upper storey canopy allowing for greater stand density and promoted aboveground light capture as well as light-use efficiency in a site as well as the complementary use of resources [2,42].

Environmental variations controlled the species richness-biomass relationships in the sampling natural systems as a causal pathway [11]. These results indicated the relationship between biodiversity and aboveground biomass are strongly dependent on variations of environment conditions, especially including of climate factors and soil disturbance [43]. In this research, our results contrast with four models including different biotic and abiotic factors. When the explanatory variables of model didn’t constitute of climate moisture index, species richness and stand age accelerated the development of aboveground biomass. Species richness just had indirect influence on the aboveground biomass after adding the climate moisture index into the models. In contrast to soil nutrient regime, climate can directly and indirectly affect and species richness and aboveground biomass through changing the species composition and community structure, and climate moisture index become a better mediation to link the relationship between species richness and aboveground biomass when we considered the effect of stand age. Generally, relative lower soil nutrient conditions were responsible for the loss of diversity and the majority of biomass production [7], and the climate moisture index increased with the soil nutrient regime which was consistent with the complementary effects by resource apply [5]. We found the rich soil nutrient regime suited more broad-leaved species due to lesser disturbance, and produced higher tree species diversity and productivity via increased resource acquisition and utilization as well as facilitation among individuals [11,44]. Relatively more species-rich systems in our study had a strong effect, negatively influenced by resource supply, with a different finding from species-poor boreal forests [11]. Further explanation is that the species composition might affect energy fluxes based on particular attributes of species that exert especially important effects on resource uptake [2]. Our finding might illustrate that a lower nutrient regime leads to broad-leaved tree species biomass loss in the mixed pine forest, but more resource utilization was allocated for *P*. *kesiya* which accumulated more biomass production in a richer soil nutrient regime with better climate conditions.

## Conclusions

Our study provides different insights into the mechanism, showing positive relationship between the species richness and the aboveground biomass in the primary *P*. *kesiya* forest. Species richness can’t affect directly the aboveground biomass through soil nutrient regime and stand age and affect indirectly the aboveground biomass through climate moisture index. The climate moisture index is crucial for the species richness-aboveground biomass relationship as a mediation variable in our study, which confirms previous studies. However, more and more sampling plot in the primary *P*. *kesiya* forest are needed for a better understanding of biodiversity effects on the forest ecosystem on a larger scale. Thus it is possible that more response variables and methods are in favour of the exploration of biodiversity-ecosystem functioning relationships in the complex forest ecosystem.

## Supporting information

S1 Table The aboveground biomass and species richness as well as stand age, soil nutrient regime and climate moisture index in each plot. (PDF)
