# Supplementary material for: The relationship between species richness and aboveground biomass in a primary Pinus kesiya forest of Yunnan, southwestern China
Source: PLoS One. 2018 Jan 11;13(1):e0191140. doi: 10.1371/journal.pone.0191140 (PMC5764369; doi:10.1371/journal.pone.0191140)
Supplement: S1 Table — (PDF) [file pone.0191140.s001.pdf]

S1 Table. The aboveground biomass and species richness as well as stand age, soil nutrient regime and climate moisture index in each plot. AGB, aboveground biomass; SP, species richness; SA, stand age; SR, soil nutrient regime; CMI, climate moisture index.

plot	AGB	SP	SA	SR	CMI
1	125.3	24	39.84	6.00	354
2	180.943	25	40.51	6.00	355
3	162.362	18	44.43	5.0	350
4	57.107	17	37.55	6.00	351
5	106.799	15	40.89	5.0	351
6	132.595	17	41.02	5.0	351
7	208.351	21	44.67	5.0	351
8	129.525	19	45.2	5.0	351
9	157.325	30	41.55	5.0	353
10	124.888	32	45.05	6.00	354
11	110.71	27	34.3	6.00	344
12	86.162	29	33.75	5.0	346
13	125.391	22	51.85	4.0	329
14	265.185	24	56.01	4.0	328
15	169.576	23	51.13	5.0	329
16	475.514	21	50.98	5.0	329
17	247.333	23	50.17	5.0	329
18	189.496	26	51.04	5.0	330
19	145.341	18	43.92	4.0	287
20	144.451	21	59.03	5.0	288
21	176.385	28	48.9	5.0	287
22	127.449	30	44.54	4.0	287
23	183.59	9	47.17	5.0	287
24	178.382	9	46.15	5.0	287
25	169.78	28	38.12	5.0	284
26	158.478	24	38.02	5.0	283
27	99.663	26	42.03	6.00	283
28	81.574	27	45.14	5.0	284
29	90.608	30	41.6	5.0	278
30	146.697	22	40.88	6.00	277
31	187.721	15	46.11	3.000	317
32	197.994	25	41.35	5.0	318
33	227.856	18	45.54	3.000	318
34	131.149	21	41.76	4.0	318
35	128.103	25	41.41	4.0	341
36	143.078	21	48.4	5.0	343

37	117.699	16	45.24	5.0	298
38	140.855	15	39.42	5.0	298
39	85.38	18	38.78	4.0	291
40	122.01	20	38.2	5.0	291
41	100.144	18	41.07	5.0	291
42	113.934	16	38.29	4.0	292
43	108.587	13	39.97	5.0	282
44	122.843	12	43.57	5.0	282
45	139.011	21	44.91	5.0	282
46	104.424	17	41.4	4.0	282
47	166.334	11	41.23	5.0	286
48	63.512	8	30.54	5.0	285
49	146.95	30	46.5	4.0	331
50	243.359	30	44.2	3.000	330
51	126.099	35	49.37	4.0	331
52	299.717	29	49.76	3.000	331
53	159.322	13	46.2	4.0	331
54	215.086	16	56.18	5.0	331
55	83.285	27	44.48	4.0	344
56	173.769	20	47.94	4.0	344
57	174.607	28	47.89	4.0	344
58	212.633	26	45.77	4.0	344
59	196.206	27	46.64	5.0	310
60	158.072	30	41.7	4.0	310
61	242.827	28	41.63	5.0	345
62	267.851	30	46.1	6.00	344
63	148.551	31	34.51	5.0	344
64	108.605	30	33.82	6.00	344
65	227.893	29	69.32	6.00	344
66	206.974	23	47.54	6.00	344
67	193.082	50	45.52	5.0	341
68	196.063	46	32.93	6.00	336
69	45.086	39	33.64	6.00	336
70	165.887	42	41.22	6.00	336
71	501.688	30	59.42	4.0	350
72	311.678	21	60.68	4.0	350
73	430.649	23	48.8	4.0	350
74	307.559	26	53.9	5.0	350
75	338.44	23	57.65	3.000	350
76	402.547	29	51.59	4.0	350
77	437.128	29	71.32	5.0	350
78	465.196	22	57.99	4.0	350
79	264.862	29	60.81	3.000	350

80	473.484	36	63.08	3.000	351
81	283.469	36	52.7	4.0	351
82	285.887	33	66.14	3.000	352
83	331.752	28	70.52	2.000	352
84	173.732	30	63.17	4.0	352
85	288.807	31	54.65	3.000	352
86	461.404	34	64.53	3.000	352
87	159.884	34	52.3	3.000	352
88	471.11	42	58.94	3.000	353
89	332.953	41	74.41	4.0	353
90	444.614	36	77.22	4.0	353
91	402.793	29	66.72	4.0	353
92	351.296	30	59.8	4.0	353
93	238.224	37	49.02	3.000	349
94	475.604	51	62.73	4.0	350
95	227.422	41	55.22	4.0	349
96	304.866	34	49.19	4.0	350
97	279.01	37	53.76	4.0	350
98	226.347	25	46.85	4.0	348
99	388.39	25	59.29	4.0	349
100	273.836	24	50.98	3.000	348
101	316.921	29	56.62	4.0	348
102	401.534	19	52.76	4.0	348
103	357.986	23	64.41	4.0	348
104	465.558	16	59.42	3.000	348
105	251.938	18	60.98	4.0	348
106	395.558	21	61.83	3.000	348
107	301.349	20	55.41	4.0	349
108	365.724	27	51.33	3.000	348
109	403.974	25	57.36	3.000	348
110	338.769	29	65.22	3.000	347
111	298.537	32	55.48	4.0	348
112	151.031	36	59.03	4.0	348
